# 

**Published:** 1964-07

**Authors:** 


### Contents

RUMINATIONS ON THE TRIGEMINAL NERVE *V. Lambert* 75

TRANSPLACENTAL FOETAL BLOOD LOSS: A COMMON EVENT . . . . *I. D. Fraser* 80

TRICKS OF THE PSYCHOTHERAPIST'S TRADE *D. Russell Davis* 83

POLYNEURITIS COMPLICATING HERPES ZOSTER *E. S. Etuk et al.* 91

PRIMARY SYSTEMIC AMYLOIDOSIS *Clinico-Pathological Conference* 93

REPORTS FROM SOCIETIES . . . . . . . 99

OBITUARY . . . . . . . . . . 100

LOCAL MEDICAL NOTES . . . . . . . 101

